# Gut Microbiota in Lung Cancer: Where Do We Stand?

**DOI:** 10.3390/ijms221910429

**Published:** 2021-09-27

**Authors:** Konstantinos Georgiou, Blagoi Marinov, Ammad Ahmad Farooqi, Maria Gazouli

**Affiliations:** 1 1st Department of Propaedeutic Surgery, Hippokration General Hospital of Athens, Athens Medical School, National and Kapodistrian University of Athens, 11527 Athens, Greece; kongeorgiou@med.uoa.gr; 2 Medical Simulation Training Center at Research Institute of Medical University of Plovdiv, Tsentar, 4002 Plovdiv, Bulgaria; bmarinov@mu-plovdiv.bg; 3 Institute of Biomedical and Genetic Engineering (IBGE), 24 Mauve Area, Sector G-9/1, Islamabad 54000, Pakistan; farooqiammadahmad@gmail.com; 4 Laboratory of Biology, Medical School, National and Kapodistrian University of Athens, 11527 Athens, Greece

**Keywords:** lung cancer, microbiome, gastrointestinal, respiratory, gut–lung axis, chemotherapy, immunotherapy

## Abstract

The gut microbiota (GM) is considered to constitute a powerful “organ” capable of influencing the majority of the metabolic, nutritional, physiological, and immunological processes of the human body. To date, five microbial-mediated mechanisms have been revealed that either endorse or inhibit tumorigenesis. Although the gastrointestinal and respiratory tracts are distant physically, they have common embryonic origin and similarity in structure. The lung microbiota is far less understood, and it is suggested that the crosslink between the human microbiome and lung cancer is a complex, multifactorial relationship. Several pathways linking their respective microbiota have reinforced the existence of a gut–lung axis (GLA). Regarding implications of specific GM in lung cancer therapy, a few studies showed that the GM considerably affects immune checkpoint inhibitor (ICI) therapy by altering the differentiation of regulatory T cells and thus resulting in changes in immunomodulation mechanisms, as discovered by assessing drug metabolism directly and by assessing the host immune modulation response. Additionally, the GM may increase the efficacy of chemotherapeutic treatment in lung cancer. The mechanism underlying the role of the GLA in the pathogenesis and progression of lung cancer and its capability for diagnosis, manipulation, and treatment need to be further explored.

## 1. Introduction

The leading cause of cancer-related deaths worldwide is lung cancer. It is associated with the highest mortality among all cancer types [1].

The human microbiota incorporates all the microorganisms that reside in all surfaces of the body that are exposed to the external environment. These include the skin, the gastrointestinal tract, the genitourinary system, and the respiratory tract. Therefore, it can be considered an environmental factor to which humans are continuously exposed in high doses throughout their lives.

The microbiota have therefore been implicated as causal or preventing factors in a variety of diseases, including cancer. This theory has been supported by rigorously controlled experiments carried out in mouse models that have been colonized with one or more specific bacteria. Moreover, there is emerging evidence that the microbiota can be manipulated in such a way in order to treat various diseases, including cancer [2].

Although the gastrointestinal and the respiratory tracts are physically distant, they have the same embryonic origin, and they have a high similarity in their structure [3]. Additionally, numerous pathways involving their respective microbiota have reinforced the existence of a gut–lung axis (GLA) [4].

In this review, we aim to focus on the role that the gut–lung axis plays in carcinogenesis and also to explore how the gut microbiome influences lung cancer, as well as the effect of the gut microbiota in the therapy response of lung cancer.

## 2. The Human Microbiome

### 2.1. Microbiota Glossary

Microbes are found on every surface of the body that is in contact with the external environment. Areas include the skin, the gastrointestinal tract, the genitourinary system, and the respiratory tract [5].

The microbiome comprises the community of symbiotic (i.e., promoting the host’s health), commensal (neutral to the host’s health, with neither beneficial nor negative effects), and pathogenic microorganisms that share our body [6]. Microbiota is the term that comprises the sum of all the species that form microbial communities. Examples include bacteria (bacteriobiota), fungi (mycobiota), viruses (virobiota), phages, archaea, protists, and helminths [7]. When it is referred to a specific environment, then the term is preceded by the respective location. Such an example is the “lung microbiota”, which refers to the respiratory tract [8].

“Microbiome” is a term that is also used, and it refers to the microbiota. The study of the genetic material deriving from all microbial DNA recovered directly from environmental samples, such as the lung, is termed metagenomics. The metagenome consists of the collective genome of the microbiota and encompasses over 100 times the number of genes of the human genome, containing approximately 10-times more genes in each microbiome [6]. “Shotgun metagenomics” is a term that can be used to describe the procedure of randomly breaking down the total DNA of a sample, followed by next-generation sequencing. Thus, primer-independent and unbiased sequencing data are generated, which thereafter can be evaluated by various reference-based and/or reference-free procedures. Therefore, shotgun metagenomics marks all the DNA material in a sample and produces abundant information for all genes, functions, and organisms [5].

Eubiosis is the term given to the condition whereby the microbiota is in a stable equilibrium, and in a healthy state. In this state, the microbiota either remains commensal or symbiotic with their hosts. However, it is rather challenging to standardize a perfect eubiosis. This is due to the large population variation. In one individual, what can be considered an optimal eubiosis may be different in another. In contrast to eubiosis, an altered microbiota structure and imbalance of the gut bacterial ecosystem is observed in various diseases, often referred to as dysbiosis [9]. Dysbiosis can augment the numbers of deleterious microbiota that generate harmful metabolites and antigens. This, in turn, can lead to maladaptive immune responses. Such interruptions are especially relevant to oncology, as they can lead to malignancies, taking into account that both decontrolled metabolism and inflammation are considered cancer traits [10].

### 2.2. Gut Microbiota under Normal Conditions

The microbial composition appears to remain fairly constant, in adult humans, under healthy conditions [11]. In the human body, the gut, the skin, and the oral cavity are the areas where microbes are found at the highest concentrations [12]. From the aforementioned areas, the most abundantly colonized organ is the gastrointestinal tract (GIT). The gut of a healthy subject is reported to contain around 1–1.5 kg of microbes. This corresponds to approximately 10^14^ bacteria (10-times more than the number of body cells) [13]. Approximately, there are 1000 species of microbes colonizing the gut. Their microbial density increases along the GI tract. In the stomach and duodenum, there can be found 10^1^–10^4^ microbes per gram; in the jejunum and ileum, 10^4^–10^8^ cells per gram; and in the colon and feces, 10^10^–10^12^ cells per gram [6].

The stomach and the small intestine contain only a small number of microbes, due to the presence of hydrochloric acid and nitric oxide, which have antimicrobial action and are found in these two organs [14,15]. The large intestine, on the contrary to the aforementioned structures, presents a better milieu for symbiotic microbes, as, after the previous digestion/absorption occurring in the small intestine, better conditions are present to retrieve energy and other essential elements from the large bowel lumen [16,17]. Although a larger amount of live microbes can be found in the colon, due to its impermeable adherent mucus layer, direct contact with the epithelium is prevented [18].

In the gut, it is estimated that there are approximately one thousand bacterial species. These contain approximately 2000 genes per species, therefore approximately yielding 2 million genes. This amounts to 100 times the number of nearly 20,000 human genes. This number is in agreement with the actual extent of microbial gene catalogues that can be found in MetaHIT and the Human Microbiome Project [19].

The structure and function of the GM throughout the entire life is influenced by many different factors, starting from birth (delivery), and with the continuation of the diet throughout childhood and adulthood, as well as from the antibiotics usage [20]. An analysis using metagenomic shotgun sequencing of the GM revealed multifactorial involvement between the microbiome and a number of extrinsic as well as intrinsic factors. These included 60 dietary factors, 31 intrinsic factors (such as blood cell counts, biochemical test, lipid concentrations), 19 drug categories, 12 diseases, and 4 smoking categories, altogether accounting for 18.7% of the inter-individual variation in the GM. Diet was found to play a significant role in altering the GM [21]. Approximately 4.5% of the BMI is estimated to be attributable to the GM [22].

The human GIT comprises a varied community of bacteria, viruses, archaea, fungi, and eukarya [23]. GM bacteria belong to two phyla. These are the *Firmicutes* (64% encompassing Gram-positive genera, e.g., *Clostridium*, *Ruminococcus*, *Lactobacillus*, *Butyrivibrio*, *Anaerostipes*, *Roseburia*, and *Faecalibacterium*) and the *Bacteriotides* (23% containing Gram-negative genera, e.g., *Bacteroides*, Porphyromonas, and *Prevotella*) [24]. The digestive tract is also occupied by other phyla. Included are the *Proteobacteria* (8% including Gram-negative genera, e.g., *Helicobacter* and Escherichia), the *Actinobacteria* (3% encompassing Gram-negative genera, e.g., *Bifidobacterium*), and less of the phyla *Fusobacteria*, *Spirochaetes*, *Verrucomicrobia* (Gram-negative species *Akkermansia muciniphila*), and *Lentisphaerae* [25]. The most dominant archaeal groups are the methanogens *(Methanobrevibacter* and *Methanosphaera*) [26,27]. Finally, accounting for less than 1% in the GM are the fungi and archaea. In the gut, the two most common fungal phyla that can be found are Ascomycota (which includes the genera *Candida* and *Saccharomyces*) and Basidiomycota [28,29]. Overall, the colon contains the highest density, with anaerobes accounting for the majority of the bacteria, such as *Bacteroides*, *Porphyromonas*, *Bifidobacterium*, *Lactobacillus*, and *Clostridium* (genera that belong to the most abundant phyla: Bacteroidetes, Actinobacteria, and Firmicutes) [30]. Two metrics are used to describe the complexity of microbiota: α and β diversity. The first one describes the abundance in a given sample (i.e., the number of the organisms and the symmetry of their distribution). The second (β diversity) defines the extent of absolute or relative overlap in shared taxa between samples [31]. Usually, a wide range of microbial β diversity exists among individuals that are enriched for a particular species, which may be minimally apparent in others [2].

The GM has its own requirements in energy. It consumes energy from the luminal contents, thereby augmenting the energy consumption [32].

Thus, the GM is considered to be the analogue of a strong “organ” capable of impacting most metabolic, nutritional, physiological, and immunological procedures of the entire human body [26,32]. The GM encompasses different genes that are involved in carbohydrate metabolism (glucose, galactose, fructose, arabinose, mannose, xylose, starch, and sucrose), consequently producing important nutrients that cannot be synthesized otherwise. Examples of these are short-chain fatty acids (SCFA) [33], vitamins (vitamin K, vitamin B_12_, folic acid), certain amino acids [34,35], neurotransmitters [36], and the regulation of gastrointestinal hormones [37,38]. Additionally, the GM produces specific enzymes capable of provoking the fermentation of indigestible carbohydrates, with 10–30% approximately of the ingested energy as other fermentation products, i.e., SCFAs (e.g., acetate, propionate, and butyrate), which are, at approximately 90–95%, absorbed in the colon and represent around 6–10% of the energy needs of the human body [39]. Some authors perceive the GM as an independent virtual organ by itself due to the abovementioned properties [40].

The human microbiome does not remain constant. It changes with age, the diet of the organism, and with its health status. The GM interacts in several ways, both in healthy and disease states in the host, and includes:

1. Modulating the inflammatory response in the host’s gut;
2. Synthesizing small molecules and proteins that are absorbed by the host;
3. Changing the quantity of obtainable energy within the diet.

In recent years, there has been an increase in research on the GI microbiota. This is essentially thanks to the important progress in phylogenetic investigation and also the quantification of GM through current high-throughput sequencing. The utilization of cost-effective, culture-independent molecular techniques on fecal samples enabled, for the first time, the accurate and reliable analysis of the dynamics of the host–GM relations. In whole-genome shotgun sequencing, the complete DNA in a given sample is fragmented, sequenced, and remapped into the original genome [41]. Then, these data are compared with the preexisting databases in order to be able to identify the different species and genes. An advantage of this method is that one is able to identify all the diverse species and all the genes that are present. This method requires a considerable amount of bioinformatic mapping, however, and is consequently considered to be computationally intensive [42,43]. In terms of the networks of genes and molecules, the Kyoto Encyclopedia of Genes and Genomes (KEGG) is a freely available knowledge base for the systematic analysis of gene functions (http://www.genome.ad.jp/kegg/, accessed on 25 September 2021). Different databases are used in order to assign functional meanings to genes and genomes. Thus, the higher-level functional changes are predicted as KEGG pathway maps [44]. However, basic research is based mostly on rodent models and cell cultures, and their significance for human physiology and clinical conditions remains unknown as limited studies have validated the interpretation of rodent-based data in an individual context in a “head-to-head” fashion [45].

The GM additionally exhibits an important role in the defense against pathogens. This is due to the fact that the large intestine poses a major challenge to the mucosal immune system. More specifically, the intestinal mucosa must be able to tolerate commensal microbiota as well as dietary antigens and eliminate pathogens successfully. Τhe GM products are critical in order to protect the host from various diseases [46] as well as shape systemic immune homeostasis [47]. In a healthy state, the GM produces antimicrobial compounds and therefore keeps the barrier intact, and it presents anti-inflammatory action, which in turn protects the epithelial cells against pathogens [20,30]. This action is mediated through toll-like receptors, which can induce the synthesis and delivery of proinflammatory factors such as tumor necrosis factor alpha (TNFα) and interleukins 1 and 6 (IL1 and IL6) [30]. The exact mechanism of this anti-inflammatory action is not well clarified. Several microbe components have been detected to increase their expansion and function, including SCFAs (especially butyrate) and polysaccharide A of Bacteroides fragilis [48].

The GM is both a producer and a consumer of vitamins: prototrophs (“producers”) are microbes that are able to synthesize vitamins de novo, in contrast to other microbes that require exogenous vitamin provision, called auxotrophs (”consumers”) [49]. Some common microbes (i.e., *Bacteroides*, *Enterococcus*, *Bifidobacterium*) display auxotrophic behavior, although they can produce most of the soluble vitamins of the B complex (cobalamin, thiamine, pyridoxine, biotin, folate, nicotinic acid, panthotenic acid) and vitamin K_2_ [50]. It must be noted, however, that the de novo biosynthesis of small micronutrient molecules demands high consumption of energy and that bacteria prefer to uptake these molecules from the environment when they are available [51].

To summarize, the GM has the capacity to satisfy the human metabolic needs, acting as an energy supplier and as a provider of certain vitamins and micronutrients to the host [49].

### 2.3. Lung Microbiota under Normal Conditions

Essential for host homeostasis and disease are the microbiotas of other sites, such as the lungs. The lung microbiota is recognized as a cornerstone in the physiopathology of numerous respiratory diseases [4].

Compared to the better-studied gut microbiota, the microbiota of the lung, considered only in recent years, represents a more discreet part of the whole microbiota associated with the human host [4]. The microbiota of the lung represents a significantly lower biomass than the gut microbiota: around 10 to 100 bacteria per 1000 human cells [52]. The lungs are heavily exposed to microorganisms due to contact with the external environment [53]. The lungs have indeed a specific microbiota, even though they were thought to be sterile in healthy individuals according to previous knowledge. The predominant bacterial phyla in the lungs, in healthy subjects, are the same as those in the gut. These are mainly Firmicutes (*Staphylococcus*, *Streptococcus*, and *Lactobacillus*) and Bacteroidetes, followed by Proteobacteria and Actinobacteria, whereas lung tumor samples tend to contain increased levels of Proteobacteria [54].

A crucial role of the lung microbiota in the maturation and homeostasis of lung immunity has been described as major inflammation in the lungs, which can drastically transform the lung microbiota composition [55].

An inter-kingdom crosstalk within the lung microbiota exists and it may involve several pathways, such as physical interaction, quorum-sensing molecules, production of antimicrobial agents, immunologic response modulation, and nutrient exchange. For instance, synergistic interactions have been documented between *Candida* and *Streptococcus*, such as the stimulation of *Streptococcus* growth by *Candida*, increasing biofilm formation, or enhancement of the *Candida* pathogenicity by *Streptococcus* [56]. However, the lung microbiota modulation is not limited to local inter-kingdom crosstalk and also depends on inter-compartment crosstalk between the gut and lungs [4].

### 2.4. The Gut–Lung Axis (GLA)

Although the gastrointestinal and respiratory tracts are physically distant from each other, they are of the same embryonic origin and they present a highly similar structure, thus implying that these two tracts might interact in a multimodal manner. Thus, a new and clear crosstalk between the respiratory and GI tracts, known as the gut–lung axis (GLA), has been established. Among the existing different inter-organ connections, the GLA remains less studied than the gut–brain axis. It has been reported that this crosstalk of the GLA organs is mediated through microbial and immune functions to achieve this two-way regulation.

Apart from gastroesophageal content inhalation and sputum swallowing, which can partially explain this inter-organ connection, the GLA also involves indirect communications: gut and lung microbes show similar colonization characteristics in the early stages of life, and the gut and lungs have a strong mucosal defense system against microbes [3]. It is reported that short-chain fatty acids (SCFAs), which are the major metabolic products of the GM from dietary fiber (especially in case of a high-fiber diet), act in the lungs as signaling molecules on resident antigen-presenting cells to attenuate the inflammatory and allergic responses [57].

The gut and lung microbiota are linked to each other by a complex dialogue between two systems through the lymphatic and blood circulation systems. For instance, the lung flora can affect the intestinal flora through the circulation of the blood [58], while the gut microbiota induces numerous respiratory diseases, such as COPD, cystic fibrosis, respiratory infection, and asthma [59]. Lately, it has been shown that intrinsic lymphoid cells, which are involved in tissue repair, are recruited from the intestine to the lungs in response to inflammatory signals provoked by IL-25 [60].

In summary, the GLA immune interplay is a bidirectional two-way process resulting from multifaceted interactions among the different microbial components of both the gut and lung microbiota, together with local and distal immune effects. Changes in this axis may lead to harmful outcomes, such as cancer development, pathogen colonization, damage of tissue, and increased susceptibility to infections [3].

## 3. Gut Microbiota in Cancer

### 3.1. Association of Pathogenic Microbes with Cancer

A common observation in many studies is that cancer cases are associated with a dysbiosis that manifests in a marked decrease in both microbial diversity and community stability. The relationship between microbial dysbiosis of pathogenic microbes and the human host appears to be considerably more complicated than initially assumed. Perhaps the best example comes from *Helicobacter pylori* infection, where the induced gastritis is strongly linked to gastric adenocarcinoma; on the other hand, *H. pylori* exhibits a protective role against Barrett’s esophagus and esophageal adenocarcinoma, possibly by affecting the stomach pH and improving gastric acid reflux [61,62].

Thus, not all subjects infected with oncogenic microbes develop cancer. The cancer prevalence and severity greatly depend on the genetic heterogeneity of both the microbiota and the host, combined with other predominant environmental parameters. For instance, only *H. pylori* strains that carry the cagA virulence factor are able to efficiently cause gastritis and gastric cancer. Additionally, another important determining factor of whether an affected individual will develop cancer is the host genetics, which may influence the immune response. Furthermore, diet and lifestyle factors such as alcohol, tobacco use, chronic inflammation, etc., play important roles as well [2].

However, it must be noted that metagenomic sequencing studies present several limitations as they cannot ascertain whether a particular microbiota change is the cause or the result of cancer. Furthermore, current 16S rRNA-based techniques lack the resolution to differentiate between commensal and pathogenic strains. Nevertheless, nowadays, whole-genome shotgun sequencing coupled with rapidly evolving bioinformatics techniques is able to resolve this limitation [63].

Very few longitudinal studies deal with the microbiota changes at different stages of tumorigenesis [2]. Furthermore, many studies have analyzed the fecal microbiome only, which is a different entity from the mucosal-associated microbiome and therefore less likely to be relevant to disease [64].

Finally, it is important to realize that the microbiota might exert oncogenic action or, in contrast, act as a tumor suppressor. Thus, a number of microbial-mediated mechanisms have been suggested that either enhance or hinder tumorigenesis. For example, to reveal the significance of the microbiota in promoting carcinogenesis, human *Escherichia coli* pks (polyketide synthase) pathogenicity strains, either pks+ or Δpks *E. coli* (containing and with deleted pks, respectively), were treated with the pro-carcinogen azoxymethane (AOM) to induce colorectal tumors. Although both *E. coli* strains stimulated inflammation to a similar extent, all of the tumors in the pks+ group became malignant, while all the tumors in the Δpks group remained benign [65]. On the contrary, for example, butyrate-producing bacteria are tumor-suppressive in vivo [66].

Five microbial-mediated mechanisms have been revealed that either promote or inhibit tumorigenesis.

#### 3.1.1. Inflammation and Immune System Alterations

It is important to recall that inflammation may present with two contradictive effects regarding tumors: while chronic, widespread inflammation is generally tumor-promoting, local inflammation restricted to the tumor microenvironment can suppress the tumor. Preclinical studies in mouse models have revealed a strong association between inflammation and colorectal cancer (CRC) mediated by the GM. The possible mechanism lies in the fact that a fiber-free diet results in increased amounts of two mucus-degrading bacteria (*Akkermansia muciniphilia* and *Bacteroides caccae*). In turn, mucus degradation leads to increased susceptibility to a mucosal pathogen, *Citrobacter rodentium*, which causes colitis, a well-known risk factor for CRC. On the other hand, researchers have observed the diminution of butyrate-producing bacteria, which promote barrier function by strengthening tight junctions between epithelial cells. Finally, it has been reported that a number of other beneficial microbes used as probiotics, including *Lactobacillus* and *Bifidobacterium*, improve barrier function and diminish permeability [67].

#### 3.1.2. Diet and Microbiota Metabolites

Several dietary components, when entering the gut, are metabolized by bacteria, producing alleged oncometabolites and/or tumor-suppressive metabolites [68]. For instance, it has been shown that high dietary intake of proteins may result in increased protein levels in the colon, where many types of bacteria, including some Firmicutes and *Bacteroides* sp., can ferment amino acids into *N*-nitroso compounds, which in turn induce DNA alkylation and mutations to the host [69].

Butyrate, a bacterial product of dietary fiber fermentation, conveys its tumor-suppressive action through diverse mechanisms. As an inhibitor of histone deacetylase, it epigenetically controls the expression of genes that participate in cell proliferation and apoptosis [66]. Additionally, it is also a ligand for a variety of colon receptors (GPR109A) that have also been implicated in tumor suppression [70]. Furthermore, butyrate plays a role in the maintenance and function of the epithelial barrier, a function that is important for preventing inflammation.

Finally, it should be emphasized that the diet dictates whether the microbiota will produce metabolites that may aggravate or ameliorate the progression of a tumor. Thus, *Clostridium scindens* produces secondary bile acids as a reaction to dietary fat, but, being also a member of *Clostridium* cluster XIVa, it generates butyrate production in response to fiber [2].

#### 3.1.3. Cell Signaling Pathways

Several signaling pathways have been described in different types of cancer. For instance, the APC tumor-suppressor gene is mutated in CRC more frequently than any other gene [71]. Enterotoxigenic *Bacteroides fragilis* (ETBF), an opportunistic pathogen associated with poor prognosis of CRC, constitutively activates STAT3 via phosphorylation and nuclear translocation in colorectal tumors [72]. Some *Salmonella typhi* strains secrete *AvrA* to activate β-catenin and are associated with hepatobiliary cancers [73].

Cellular signaling pathways may also modify different bacterial virulence factors. Thus, the *H. pylori* cagA, being an important virulence factor, is extensively phosphorylated by cellular Src and Abl kinases. Unphosphorylated and phosphorylated CagA have different interactions with a broad repertoire of cellular signaling proteins, many of which are involved in regulating cellular proliferation pathways [74].

#### 3.1.4. DNA Damage

It is well known that DNA damage is a major factor causing carcinogenesis. The mechanisms of action by which the genotoxins directly exert their damaging effects on host cell DNA involve either forming adducts or initiating double-stranded breaks in DNA, which, when unresolved by normal DNA repair processes, may lead to mutations, insertions, deletions, or chromosomal inversions and translocations. The cytolethal distending toxin (CDT) produced by certain proteobacteria also provokes similar DNA damage [75]. The metabolites produced from the GM can also exert an indirect genotoxic action, by producing free radicals and affecting reactive oxygen species (ROS). Additionally, it has been shown that bile acids precipitously induce ROS as well as reactive nitrogen species (RNS) together, which can then damage host cell DNA [76].

#### 3.1.5. Distant Sites

The GM, metabolites, and immune cells that reside in the intestine can escape from it through the circulation and thus impact tumorigenesis in distant sites of the body. Before entering the systemic circulation, they arrive through the enterohepatic circulation and hepatic portal vein, reaching the liver. Therefore, the liver plays the role of gatekeeper for the identification of potentially harmful endobiotic and xenobiotic compounds and thus exercises an important role in normal body physiology as well as in gut and extra-intestinal diseases.

Other GM-derived byproducts associated with cancer prevention, such as equol, have been identified in a range of tissues (e.g., breast) as well as in several biological fluids, such as blood, urine, and prostatic fluid [77]. Furthermore, since it has been found that the GM participates in the metabolism of endogenous estrogens, it is thus associated with breast cancer [78]. Additionally, the gut response to inflammation has been shown to affect breast cancer progression [79].

Lastly, it is necessary to emphasize the synergistic action that each of the above mechanisms exhibits with the others. For instance, the chronic inflammation of IL-10 knockout mice apparently increases pks oncogenesis. Such combined action mechanisms might promote oncogenesis after an initiating event that otherwise may not be sufficient to drive transformation in isolation.

## 4. Gut Microbiota in Lung Cancer

Lung cancer (LC), with two types, namely small-cell lung cancer (SCLC) and non-small-cell lung cancer (NSCLC), is one of the deadliest malignancies in the world [80]. As NSCLC represents the majority of LC cases, knowledge of the mechanisms by which the microbiome may affect its progression is vital to improve patients’ survival and treatment responses. Compared to the GI tract, the lung microbiota is poorly understood and it is suggested that the crosslink between human microbiome and lung cancer is a complex multifactorial relationship [81].

Usually, LC patients are infected with Firmicutes, Bacteroidetes, and Proteobacteria [82], including genera such as *Granulicitella*, *Streptococcus*, *Veillonella*, and *Mycoplasma* [83]. It has been reported that Gram-negative bacteria such as *Haemophilus influenzae*, *Enterobacter* spp., and *Escherichia coli* also tend to inhabit LC [84]. Regarding the gut microbiota, it is worth noting that a lower concentration of Firmicutes and Proteobacteria, combined with relatively higher levels of Bacteroidetes and Fusobacteria, has been detected in LC patients as compared to healthy individuals [85]. It appears that these phyla are consistently found, irrespectively of microbial changes in cancer. A summary of published articles regarding microbiota changes in lung cancer patients is presented in Table 1.

Chronic infection of the lungs may be the initiating cause of cancer when microbiota dysbiosis results in a more hypoxic, tumor-promoting environment [54]. Furthermore, an increase in anaerobic respiration is observed in LC, due to the elective anaerobic qualities of the bacteria that preferentially colonize tumors. As LC progresses, these bacteria increase in number, further enhancing the hypoxic, and proinflammatory tumor environment [54]. Additionally, besides tumor involvement, the impact of cancer therapy on the pathogenic microbiome is also increasing [96].

The GLA is considered a bidirectional connection that connects the GI tract microbiome with that of the lungs, with changes in one tissue affecting the other, a key regulator being the translocation of the gut microbiota and its products across the epithelial barrier and then into the bloodstream [59]. Additionally, translocation stimulates a toll-like receptor (TLR) response and subsequent T cell expansion in distant tissues [97]. Translocation of bacteria from the GI tract can enhance tumor-specific responses through TLRs, or through the induction of memory responses, as observed for relations between *Enterococcus hirae* and small-cell lung cancer (SCLC) [98].

## 5. Gut Microbiota in Lung Cancer Therapy

### 5.1. Gut Microbiota in Cancer Treatment

Several preclinical and clinical studies in humans have shown that the GM can modify the host response to a plethora of anticancer regimens, mainly through immunomodulation. It appears that dysbiosis is not only the consequence but often also the reason for the observed variance in responses to therapy.

#### 5.1.1. Chemotherapy

Eliminating the microbiota by the administration of broad-spectrum antibiotics substantially alters the host gene expression: genes promoting cancer metabolism and progression become upregulated, with a simultaneous downregulation in inflammatory, phagocytic, and antigen-presenting paths. Nevertheless, broad-spectrum antibiotics administration can eradicate a large amount of GI commensals and thus provide opportunities for pathogens such as *Clostridium difficile* to flourish. Furthermore, treatment with antibiotics decreases the recruitment of immune cells, which are of importance for intermediating tumor regression through a corresponding decrease in their proinflammatory action [2].

It is evident that chemotherapy modifies the patients’ microbiota composition, although the impact of this alteration regarding prognosis remains unclear [99]. Additionally, and more importantly, the specific microbial composition can affect the response of a variety of conventional chemotherapeutic agents, as has been shown in studies conducted in mouse models [45].

For instance, cyclophosphamide (CP), a widely used chemotherapy agent, diminishes the villus height in the small intestine and interrupts the intestinal barrier, thus translocating the commensals towards secondary lymphoid organs, and, at the same time, it causes the accumulation of inflammatory cells. Moreover, it has been shown that antibiotics that selectively target Gram-positive bacteria, when compared to Gram-negative antibiotic therapy, significantly reduce CP’s effectiveness. Therefore, specific Gram-positive bacteria (*Lactobacillus johnsonii*, *L. murinus*, *Enterococcus hirae*, and segmented filamentous bacteria) have been found to be essential to mediate CP’s antitumor action [100].

Oxaliplatin induces its tumor retardation action through a microbiota-dependent path, as its efficacy depends on the intratumoral production of reactive oxygen species (ROS), which in turn become reduced with reduced intratumoral DNA damage [101]. This suggests that the microbiota-mediated immunomodulatory effects in response to chemotherapy compounds blur the distinction between chemotherapy and immunotherapy.

It has also been reported that the prevalence, severity, and treatment of cancer can be altered by the number of specific bacteria present or absent. For example, patients undergoing immunotherapy may benefit from *B. intestinihominis* or *E. hirae* species to improve the efficacy [102], while patients receiving irinotecan may benefit from bacterial β-glucuronidase-targeting drugs [103].

Interestingly, in patients with advanced lung and ovarian cancer with increased levels of *E. hirae* and *B. intestinihominis*, specific Th1 cell memory responses were predicted to have lengthened progression-free survival. Following this evidence, it has been suggested to add particular Enterococcus and Barnesiella species into an optimized microbiota cocktail concurrently administered with CP as well as with other alkylating agents. In the future, these bacteria or their specific immunomodulatory products/metabolites could be incorporated as adjuvants to increase the effectiveness of existing chemotherapeutics [45].

#### 5.1.2. Immunotherapy

It has been shown that commensal microorganisms are required for the maturation, function, and adjustment of the immune system. Additionally, a close and continuous interaction of immune cells with microorganisms allows for an exploration of the difference between commensal and pathogenic bacteria [2]. Numerous studies have already shown that the GM regulates the potential of immunotherapy to stimulate the anticancer immune response [45].

Monoclonal antibodies against CTLA-4 (ipilimumab), PD-1 (nivolumab), and PD-L1 (pembrolizumab) are immune checkpoint inhibitors (ICIs) that induce the individual immune response of a patient against a tumor. It has been shown that these monoclonal antibodies are highly effective for treating different types of cancer (melanomas, Hodgkin’s lymphoma, lung, kidney, bladder cancer, etc.).

It appears that the effectiveness of immune checkpoint inhibitors is dependent on the patient’s GM, which in turn closely interacts with the patient’s immune system. Thus, this interaction between the GM and ICI may explain the reported interindividual variation in patients’ responses to ICIs [104].

ICI administration usually has gastrointestinal and hepatic complications, such as hepatitis, diarrhea, and enterocolitis, as a result of a complicated interchange mechanism between host genetics, immune responses, the environment, and the microbiota [105].

#### 5.1.3. Microbial Drug Targets in Oncology

Microbial drug targets may possess the capacity to enhance the side effects of many chemotherapeutic regimens. Certain side effects, such as those provoked by irinotecan (camptothecin), may so severe that it is necessary to constrain the dose or the therapy duration [45]. Irinotecan is metabolized into an active chemotherapeutic agent (SN38) and blocks DNA replication by rapidly dividing cells and therefore is used for colorectal and pancreatic cancer treatment. The microbiota provokes increased SN38 levels in the intestine, which may trigger severe diarrhea. Furthermore, it has been shown that germfree mice exhibit less GI damage and therefore tolerate higher doses of irinotecan compared to conventional mice with an intact microbiota [106].

Furthermore, some chemotherapeutics, such as doxorubicin, have action that is similar to that of irinotecan agents and therefore can cause adverse effects in the gastrointestinal and the respiratory tract [107]. Therefore, it is suggested that targeting the microbiota may diminish the toxicity of many chemotherapeutic agents.

### 5.2. Gut Microbiota in Lung Cancer Treatment

The various effects of the gut microbiota on cancer therapy for NSCLC are presented in Table 2.

The characteristics of the GM in lung cancer patients vary widely, suggesting that the gut microbiota may affect lung cancer prognosis and therapy [3].

The implications of the specific GM in cancer therapy have been discovered by assessing drug metabolism directly and by assessing the host immune modulation response [111]. It has been shown that the GM significantly affects immune checkpoint inhibitor (ICI) therapy by altering the differentiation of regulatory T cells and thus resulting in changes in immunomodulation mechanisms [112]. The same authors found that *Akkermansia muciniphila* positively responds to ICI treatment. Supplementation with *Akkermansia muciniphila* increased the response to ICI, whereas an abnormal composition of the GM is associated with resistance to ICI treatment [112]. The GM in lung cancer patients responding to treatment with ICIs significantly differs when compared with that of patients who do not respond to immunotherapy. Furthermore, it was found that a significantly higher response to anti-PD1 therapy in lung cancer patients was positively correlated with the abundance of *Akkermansia muciniphila* species [3].

A recent work showed that the diversity of the gut microbiota in fecal (*Proteobacteria*, *Firmicutes*, *Bacteroidetes*, and *Actinobacteria*) bacteria increased the response to anti-PD-1 immunotherapy [93]. Furthermore, a previous study stated that, in NSCLC patients who responded to nivolumab, the GM composition was relatively stable and higher diversity was noted. Moreover, extended progression-free survival (PFS) has been shown in patients with high microbiome diversity as compared to those presenting low diversity [113].

A retrospective evaluation study of 118 patients suffering from advanced NSCLC and receiving immune checkpoint blockage showed that adding supplementary therapy with *Clostridium butyricum* (CBT) before and/or after the immune checkpoint blockade therapy resulted in significantly prolonged progression-free survival and overall patient survival [114].

In addition to the observed improvement of the response to ICI therapy, the GM also affects the efficacy of chemotherapeutic treatment in lung cancer. For instance, it was shown that the per os intake of *Lactobacillus acidophilus* during cisplatin treatment in lung cancer mouse models enhanced cisplatin’s antitumor efficacy, reduced the tumor size, and improved the survival rate. These findings suggest that the coadministration of probiotics improves the anti-growth and pro-apoptotic effects of cisplatin [115]. Additionally, patients with end-stage lung cancer and undergoing chemo-immunotherapy, who additionally received *Enterococcus hirae* and *Barnesiella intestinihominis*, showed a longer PFS [102]. Therefore, the increased survival of these patients may be attributed to the improvement of the immunomodulatory effect.

Additionally, studies have shown that high consumption of yogurt is beneficial as it has been shown to cause a significant reduction in lung cancer risk by 30%, implying that prebiotics and probiotics might have a protective effect in lung carcinogenesis [116].

Finally, it has been shown that a change in gut diversity could potentially be used as an indicative biomarker for the diagnosis and treatment of lung cancer [117]. However, the role of the GM in the development and progression of lung cancer needs further exploration. Furthermore, the potential actions of the microbiome in the effective modulation of anticancer treatment should be further explored and evaluated.

## 6. Conclusions

The GLA has recently emerged as an intensive two-way dialogue between the lungs and the gut, involving both in a bidirectional process, with microbial and immune interactions. Each organ and each kingdom compartment play an important role in this two-way dialogue and thus influence the host’s health. The mechanism underlying the role of the GLA in the pathogenesis and progression of lung cancer, and its capability for the diagnosis, manipulation, and treatment of lung cancer, need to be further explored.

To achieve this, randomized controlled clinical trials should be conducted with improved methodologies to determine the clinical value of the microbiota–cancer relationship and illuminate the mechanisms involved in how lung cancer is affected by the microbiome in order to discover new diagnostic and therapeutic approaches. There is also the potential to use lung and gut microbes as biomarkers for the assessment of the progression and the efficacy of the treatment of lung cancer, as well as to discover alternative methods for cancer prevention. It can also be anticipated that “design probiotics” and other methods capable of flora regulation and management could enhance the beneficial, curative action as well as the prognosis of lung cancer patients.

## Figures and Tables

**Table 1:** Relationship between microbiota and lung cancer.

Ref	Phylum	Genus	Species	Sample Type	Amount in Control Samples	Amount in Cancer Samples
[86]	Firmicutes	*Staphylococcus*	*S. epidermidis*	LC tissue biopsies	N/A	25%
[86]	Firmicutes	*Streptococcus*	*S. mitis*	LC tissue biopsies	N/A	21.87%
[87]	Firmicutes	*Streptococcus*	*S. pneumoniae*	BAL	7.30%	15.17%
[88]	Firmicutes	*Streptococcus*	Not specified	BWF	N/A	12%
[89]	Firmicutes	*Veillonella*	Not specified	BAL of LC patients (vs. patients with benign masses)	4%	11.4%
[88]	Firmicutes	*Veillonella*	Not specified	BWF	N/A	8%
[90]	Actinobacteria	*Enterococcus*	Various	Stool	0.23%	4.26%
[90]	Actinobacteria	*Bifidobacterium*	Various	Stool	4.7%	1.51%
[85]	Bacteroidetes, Fusobacteria, Cyanobacteria, Spirochaetes, Lentisphaerae		Not specified	Stool	N/A	Statistically higher
[85]	Firmicutes, Proteobacteria	*Escherichia-Shigella, Kluyvera, Faecalibacterium, Enterobacter, Dialister*	Not specified	Stool	N/A	Statistically lower
[91]	Butyrate-producing bacteria	*Faecalibacterium prausnitzii*, *Clostridium leptum*, *Ruminococcus, Roseburia* spp.	*Clostridial cluster, Clostridial cluster XIVa*	Stool	Various	From *p* < 0.0001 to *p* = 0.035
[92]	Bacteroides, Proteobacteria	*Enterobacteriaceae*, *Akkermansia muciniphila*	*Ruminococcus*	Stool	N/A	Statistically higher
[93]	Bacteroidetes, Firmicutes, Proteobacteria, Actinobacteria		*15 species*	Stool	N/A	Somehow higher
[94]	Firmicutes, Bacteroidetes, Proteobacteria	*Enterobacteriaceae*, *Streptococcus*, *Prevotella*	Not specified	Stool	N/A	Statistically higher
[95]	Bacteroidetes, Proteobacteria	*Rikenellaceae*, *Prevotella*, *Streptococcus*, *Lactobacillus*, *Bacteroides plebeius*, *Oscillospira*, *Enterobacteriaceae*	Not specified	Stool	N/A	Statistically higher

LC: Lung cancer, BAL: Bronchoalveolar lavage, BWF: Bronchial washing fluid, N/A: Not available.

**Table 2:** Potentiating or inhibitory effects of microbiota on cancer therapy for NSCLC.

Treatment	Bacteria	Enhancing or Inhibiting	Effects	Ref
Cisplatin	*Lactobacillus Bifidobacterium*	Enhancing	Decrease in oncogenic VEGF and Ras levels.	[108]
Gemcitabine	*Mycoplasma Gammaproteobacteria* *(E. coli)*	Inhibiting	Bacterial CDA metabolizes nucleoside analogues and reduces efficacy.	[109,110]
Ipilimumab	*B. fragilis*	Enhancing	Aid in tumor-specific cytotoxic T cell expansion to promote tumor-specific response.	[98]
Anti-PD-1	*B. fragilis* *A. muciniphila*	Enhancing	Aid in tumor-specific cytotoxic T cell expansion to promote tumor-specific response.	[97]

## Data Availability

Not applicable.
